# DNA Methyltransferase Inhibitor Zebularine Inhibits Human Hepatic Carcinoma Cells Proliferation and Induces Apoptosis

**DOI:** 10.1371/journal.pone.0054036

**Published:** 2013-01-08

**Authors:** Kazuaki Nakamura, Kazuko Aizawa, Kazuhiko Nakabayashi, Natsuko Kato, Junji Yamauchi, Kenichiro Hata, Akito Tanoue

**Affiliations:** 1 Department of Pharmacology, National Research Institute for Child Health and Development, Tokyo, Japan; 2 Department of Maternal-Fetal Biology, National Research Institute for Child Health and Development, Tokyo, Japan; University of North Carolina School of Medicine, United States of America

## Abstract

Hepatocellular carcinoma is one of the most common cancers worldwide. During tumorigenesis, tumor suppressor and cancer-related genes are commonly silenced by aberrant DNA methylation in their promoter regions. Zebularine (1-(β-_D_-ribofuranosyl)-1,2-dihydropyrimidin-2-one) acts as an inhibitor of DNA methylation and exhibits chemical stability and minimal cytotoxicity both *in vitro* and *in vivo*. In this study, we explore the effect and possible mechanism of action of zebularine on hepatocellular carcinoma cell line HepG2. We demonstrate that zebularine exhibits antitumor activity on HepG2 cells by inhibiting cell proliferation and inducing apoptosis, however, it has little effect on DNA methylation in HepG2 cells. On the other hand, zebularine treatment downregulated CDK2 and the phosphorylation of retinoblastoma protein (Rb), and upregulated p21^WAF/CIP1^ and p53. We also found that zebularine treatment upregulated the phosphorylation of p44/42 mitogen-activated protein kinase (MAPK). These results suggest that the p44/42 MAPK pathway plays a role in zebularine-induced cell-cycle arrest by regulating the activity of p21^WAF/CIP1^ and Rb. Furthermore, although the proapoptotic protein Bax levels were not affected, the antiapoptotic protein Bcl-2 level was downregulated with zebularine treatment. In addition, the data in the present study indicate that inhibition of the double-stranded RNA-dependent protein kinase (PKR) is involved in inducing apoptosis with zebularine. These results suggest a novel mechanism of zebularine-induced cell growth arrest and apoptosis via a DNA methylation-independent pathway in hepatocellular carcinoma.

## Introduction

Hepatocellular carcinoma (HCC) is the sixth most common newly diagnosed cancer and the third most common cause of cancer mortality worldwide. Its treatment outcome is far from satisfactory and the five-year survival rate is dismal (approximately 10%) [1]. Liver transplantation is currently considered to be the only curative therapy. Unfortunately, however, a majority (>80%) of patients with advanced and unresectable HCC are not suitable candidates for transplantation or surgical resection [2], [3]. Chemotherapy using conventional cytotoxic drugs, such as doxorubicin, cisplatin, and fluorouracil, is a common treatment option, especially for patients with unresectable tumors. However, because of poor response rates, severe toxicities, and high recurrence rates, the mean survival time is approximately six months [3], [4]. Thus, there is a very high demand for more effective agents to better combat this malignancy.

It has been considered that hypermethylation of CpG islands in tumor suppressor genes represents one of the hallmarks in human cancer development [5], [6]. It has been reported that the analysis of gene expression and promoter CpG island hypermethylation in HCC revealed that both genetic and epigenetic changes contribute to the initiation and progression of liver cancer and are correlated with poor survival [7]. Epigenetic changes such as DNA methylation are pharmacologically reversible, and this offers a promising multi-target translational strategy against cancer in which the expression of a variety of silenced genes could be reactivated. DNA methylation is specifically mediated by the action of DNA methyltransferase (DNMT) enzymes [8], which includes DNMT1, DNMT2, DNMT3a, and DNMT3b [9]. DNMT1 has de novo as well as maintenance methyltransferase activity, and DNMT3a and DNMT3b are potent de novo methyltransferase [10]. Overexpression of DNMT has been reported to be involved in tumorigenesis [11] and has been suggested as a prognostic factor in large B cell lymphomas [12]. Therefore, it has been proposed that the inhibition of DNMT activity can strongly reduce the formation of tumors [13]. Thus far, three DNMT-inhibiting cytosine nucleoside analogs (i.e., 5′-azacitidine, decitabine, and zebularine) have been studied as potential anti-cancer drugs [14]–[16]. Decitabine and its prodrug 5′-azacitidine are two widely used DNMT inhibitors for the treatment of patients with various cancers, such as myelodysplastic syndromes (MDS) and acute myeloid leukemia (AML) [17], [18]. Although Decitabine and its prodrug 5′-azacitidine are effective in treating various cancers [17], [18], the formation of irreversible covalent adducts with DNA may cause long-term side effects, including DNA mutagenesis, a potential cause of tumor recurrence.

Zebularine is a second-generation, highly stable hydrophilic inhibitor of DNA methylation with oral bioavailability that preferentially targets cancer cells [19], as demonstrated in bladder, prostate, lung, colon, and pancreatic carcinoma cell lines [20]. It acts primarily as a trap for DNMT protein by forming tight covalent complexes between DNMT protein and zebularine-substitute DNA [21]. Zebularine is also a cytidine analog that was originally developed as a cytidine deaminase inhibitor. It exhibits low toxicity in mice, even after prolonged administration [22]–[24]. Given that aberrant methylation is a major event in the early and late stages of tumorigenesis [25], [26], including hepatocarcinogenesis [7], this process may represent a critical target for cancer risk assessment, treatment, and chemoprevention [19]. In the previous study, a zebularine signature that classified liver cancer cell lines into two major subtypes with different drug response was identified. In drug-sensitive cell lines, zebularine caused inhibition of proliferation coupled with increased apoptosis, whereas drug-resistant cell lines were associated with the upregulation of oncogenic networks (e.g., E2F1, MYC, and TNF) [19]. However, little is known about the anti-cancer effect and possible mechanism of action of zebularine on HCC.

In the present study, we investigated the molecular mechanism of zebularine against HCC. We demonstrated that zebularine exhibited antitumor activity by inhibiting cell proliferation and inducing apoptosis. This effect was independent of DNA methylation, and characterized by the downregulation of CDK2 and the phosphorylation of retinoblastoma protein (Rb) as well as the upregulation of p21^WAF/CIP1^ and p53. We also found that zebularine induced apoptosis though the intrinsic and extrinsic apoptosis pathways. In addition, the data in the present study suggest that the inhibition of the double-stranded RNA-dependent protein kinase (PKR) is involved in inducing apoptosis with zebularine.

## Materials and Methods

### Cell culture

HepG2 cells (JCRB1054) and HeLa cells (JCRB9004) were purchased from the Health Science Research Resources Bank (Japan Health Sciences Foundation, Osaka, Japan), and were maintained at 37°C under an atmosphere of 95% air and 5% CO_2_ in Dulbecco's modified Eagle's medium (DMEM) containing 10% fetal bovine serum (FBS), 100 U/ml penicillin, and 100 μg/ml streptomycin. Cells were immersed in a culture medium containing the indicated zebularine concentrations. Zebularine (Wako Pure Chemical Industries, Osaka, Japan) was dissolved in distilled water as a stock solution.

### Cell viability assay

The cell viabilities after exposure to zebularine were determined using WST assay. The assay was performed using a Cell Counting Kit-8 (Dojindo Laboratories, Kumamoto, Japan) according to the manufacturer's instructions. Cell cultures exposed to 0 μM zebularine were considered to be 100% viable. The cell viability of each drug-treated sample was presented as a percentage of the viability of cultures treated with 0 μM zebularine. All samples were run five times in the same assay.

### Apoptosis analysis

Quantification of apoptotic cells was performed using a Cell Death Detection ELISA^PLUS^ (Roche Diagnostics, Tokyo, Japan). After 72 h of incubation with zebularine, cells were lysed with a lysis buffer (included in the kit). The assay was performed according to the manufacturer's instructions. Absorbance values were measured at 405 nm using a microplate reader (ARVO, PerkinElmer Japan, Kanagawa, Japan). The apoptotic ratio of each drug-treated sample was presented as a fold-change of the apoptosis of cultures treated with 0 μM zebularine. All samples were run five times in the same assay.

### 5-bromo-2′-deoxy-uridine (BrdU) incorporation assay

Cellular DNA synthesis rates were determined by measuring BrdU incorporation with the commercial Cell Proliferation ELISA System (Roche Diagnostics). After 24 h of incubation with zebularine, cells were incubated for 3 h with a BrdU labeling solution (included in the kit) containing 10 μM BrdU. The assay was performed according to the manufacturer's instructions. Absorbance values were measured at 405 nm using a microplate reader. The BrdU incorporation of each drug-treated sample was presented as a percentage of the BrdU incorporation of cultures treated with 0 μM zebularine. All samples were run five times in the same assay.

### Illumina Infinium HumanMethylation450 BeadChip analysis

Genomic DNA was extracted from three independent cell culture batches for zebularine (1000 μM)-treated and control HepG2 cells. Genomic DNA (1000 ng) was bisulfite-treated and purified using the EpiTect Bisulfite Plus Kit (QIAGEN K.K., Tokyo, Japan). Three hundred nanograms of bisulfite-treated DNA were hybridized to the Illumina Infinium HumanMethylation450 BeadChip using Illumina-supplied reagents and protocols. Both the CpG loci included on this array and the technologies behind the platform have been described previously [27]. GenomeStudio software (Illumina) was used to calculate the methylation level at each CpG site as beta value (β  =  intensity of the methylated allele [M]/[intensity of the unmethylated allele (U) + intensity of the methylated allele (M) + 100]) [27]. Region-level methylation analysis was conducted using the IMA package [28].

### Caspase assays

Caspase-3/7, -8, and -9 activities were assayed with Caspase-Glo Assays (Promega KK, Tokyo, Japan) according to the respective manufacturer's standard cell-based assay protocol. The luminescence of each sample was measure using a plate-reading luminometer. Comparison of the luminescence from a treated sample with a control sample enables determination of the relative increase in caspase activity. All samples were run five times in the same assay.

### Overexpression of PKR and forward transfection

The PKR plasmid, pFN21A-hPKR (pFN21AE2332), and empty vector, HaloTag control vector, were purchased from Promega. Transient transfection in HepG2 cells was performed according to the Lipofectamine 2000 (Invitrogen, LifeTechnologies Japan, Tokyo, Japan) methods. Cells cultured in a six-well culture plate were washed twice with phosphate-buffered saline and the medium was replaced with 2 ml of Opti-MEM (Invitrogen) with 1% FBS. Two micrograms per well of pFN21A-hPKR or the empty vector (HaloTag control vector) were then mixed with 10 μl/well of Lipofectamine 2000 in Opti-MEM and the mixture was added to the wells 20 min later. After 6 h of transfection, the cells were then cultured in regular medium for 48 h and subsequently treated with zebularine for 72 h.

### Immunoblotting

Cells were lysed in lysis buffer (20 mM HEPES–NaOH pH 7.5, 150 mM NaCl, 1% NP-40, 1.5 mM MgCl2, 1 mM EGTA, 1 μg/ml leupeptin, 1 mM PMSF, and 1 mM Na_3_VO_4_) and stored at −80°C until use. After centrifugation, aliquots of the supernatants underwent sodium dodecyl sulfate polyacrylamide gel electrophoresis (SDS-PAGE). The electrophoretically separated proteins were transferred to polyvinylidene fluoride (PVDF) membranes, blocked, and immunoblotted with anti-CDK2 (78B2, #2546), Rb (4H1, #9309), phospho-Rb (Ser807/811) (#9308), p21^WAF/CIP1^ (12D1, #2947), p44/42 mitogen-activated protein kinase (MAPK) (137F5, #4695), phospho-p44/42 MAPK (The202/Thy204) (#4370), Bax (D2E11, #5023), Bcl-2 (50E3, #2870), PKR (N216, #2766), DNMT1 (D63A6, #5032) (Cell Signaling Technology Japan, Tokyo, Japan), phospho-PKR (E120, ab32036, abcam, Tokyo, Japan), p53 (M 7001, Dako Japan, Tokyo, Japan), DNMT3a (sc-20703), DNMT3b (sc-81252) (Santa Cruz Biotechnology, Santa Cruz, CA), or glyceraldehyde 3-phosphate dehydrogenase (GAPDH) (#MAB374, Millipore, Temecula, CA) antibodies, and then with peroxidase-conjugated secondary antibodies (NA931 or NA940, GE Healthcare Japan, Tokyo, Japan). The bound antibodies were detected using the ECL system (GE Healthcare Japan).

### Statistics

All experiments were performed at least three times. Values are expressed as means ± standard error of the mean (SEM). Statistical analyses were performed using an unpaired Student's *t*-test or two-way analysis of variance (ANOVA) followed by Fisher's protected least significant difference as a post-hoc test. *p*<0.05 was considered to indicate statistical significance.

## Results

### The effects of zebularine on HepG2 cell viability

**Figure 1:**
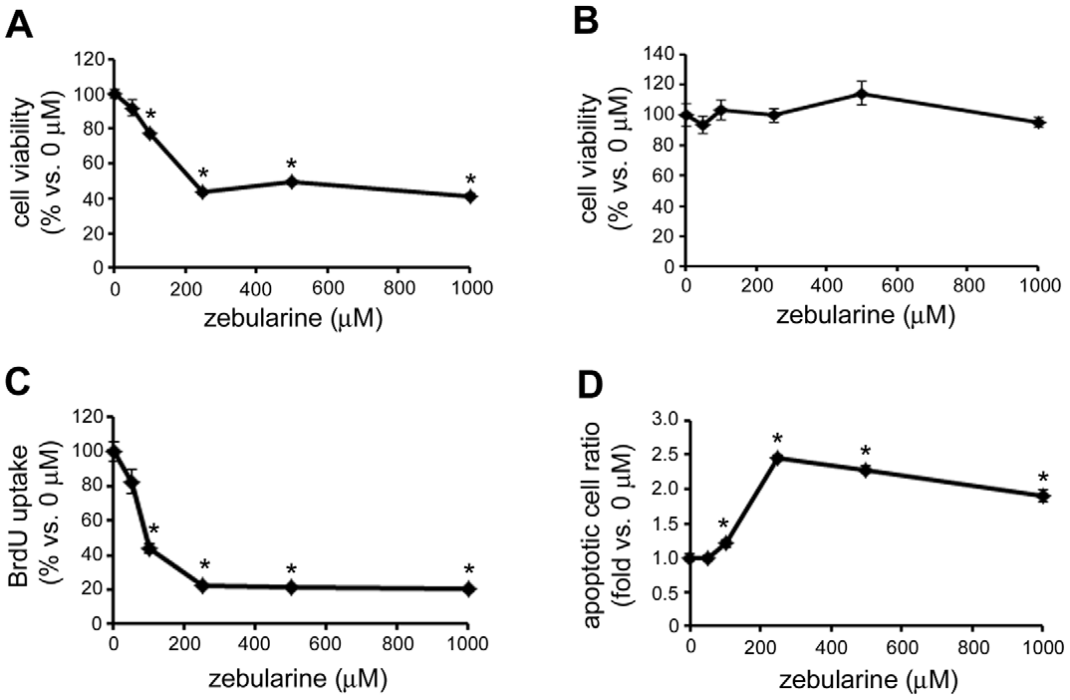
The effect of zebularine on HepG2 cell viability. HepG2 cells were treated with zebularine at indicated concentrations for 72 h (A) and 24 h (B). Cell growth was measured by WST assay. (C) HepG2 cells were treated with zebularine at indicated concentrations for 24 h. Uptake of BrdU was measured by ELISA. (D) HepG2 cells were treated with zebularine at indicated concentrations for 72 h. Apoptosis was measured by TUNEL assay. Data are the means ± SEM of results from at least three independent experiments. *p<0.05, compared to 0 μM.

In order to investigate the effect of zebularine on HepG2 cell viability, we performed WST assay after zebularine exposure. WST assay indicated that zebularine affected cell viability. Exposure of cells to zebularine for 72 h resulted in a decrease in cell viability (Fig. 1A). To further determine whether zebularine could inhibit the proliferation of HepG2 cells, we conducted BrdU incorporation assay after zebularine treatment for 24 h. Although WST assay indicated that zebularine could not affect cell viability after 24 h (Fig. 1B), BrdU incorporation assay clearly showed that the uptake of BrdU by HepG2 cells was already reduced after 24 h exposure to zebularine (Fig. 1C). At a concentration of 250 μM, the uptake of BrdU was reduced to 22.1±0.6% compared with 0 μM and a similar reduction of BrdU uptake (20.1±1.5%) was observed at a concentration of 1000 μM. In addition, we examined whether zebularine could induce HepG2 cell death. Terminal deoxynucleotidyl transferase dUTP nick end labeling (TUNEL) assay demonstrated that zebularine induced apoptotic cell death on HepG2 cells. Exposure of cells to zebularine for 72 h resulted in an increase in the number of apoptotic cells (Fig. 1D). These results indicated that DNA replication was blocked and apoptotic cell death was induced by treatment with zebularine, which resulted in reduced HepG2 cell viability.

### Zebularine affects HepG2 cells growth arrest and apoptosis via DNA methylation-independent pathway

**Figure 2:**
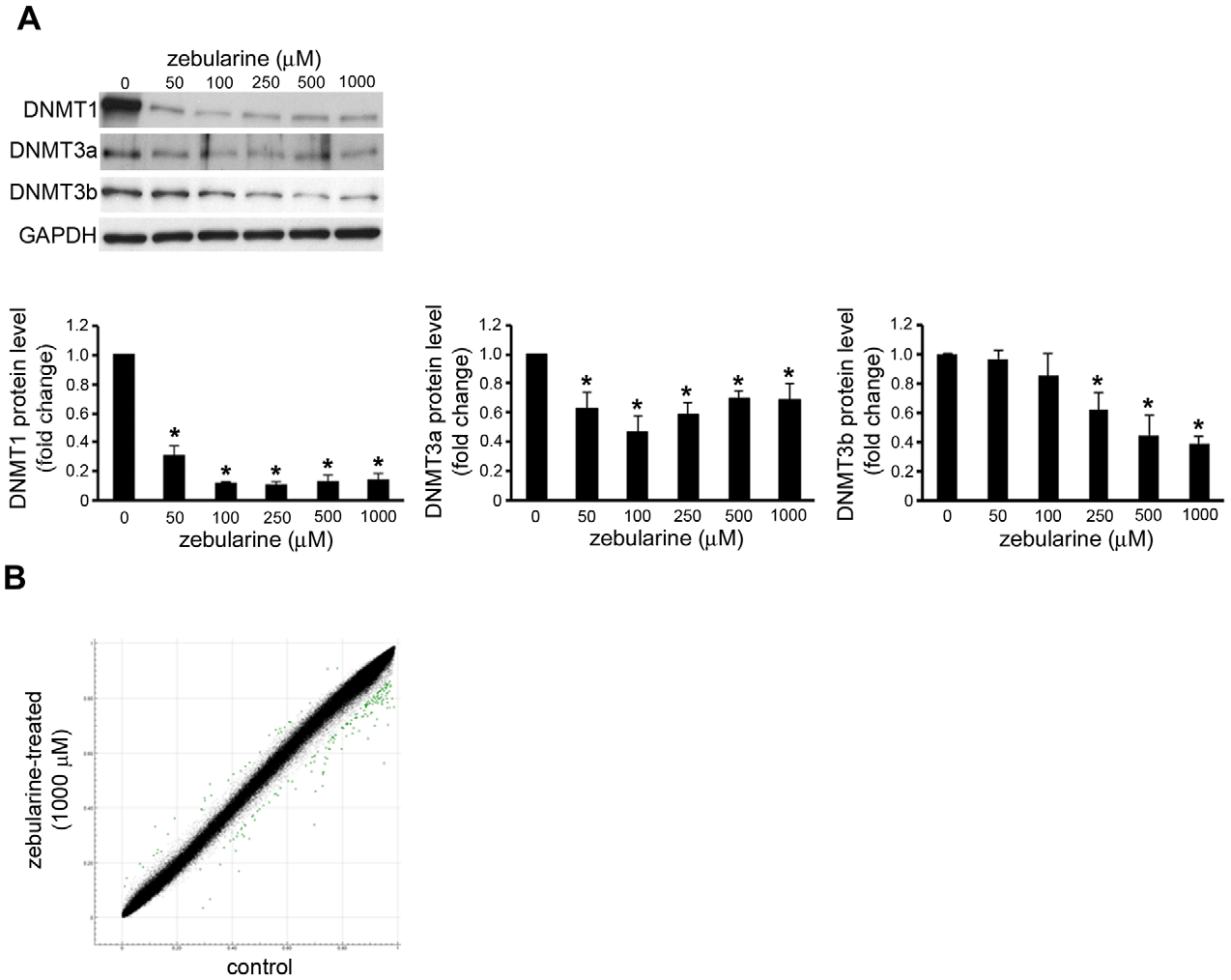
Effect of zebularine on the DNMTs expression and DNA methylation in HepG2 cells. (A) The protein level of DNMT1, DNMT3a, and DNMT3b after zebularine treatment for 72 h at different concentrations. After treatment, the cells were harvested and western blot analysis was performed to detect the protein level of DNMT1, DNMT3a, and DNMT3b. GAPDH was used as a loading control. Data are the means ± SEM of results from at least three independent experiments. *p<0.05, compared to 0 μM. (B) Scatter plot of the average beta values at 485,415 CpG sites for zebularine-treated (y-axis) and control (x-axis) HepG2 cells (n = 3 for each group). Dots for CpG sites whose delta-beta value is >0.1 or <−0.1 are shown in green (35 [0.0072%] hypermethylated and 162 [0.033%] hypomethylated CpG sites).

Because of zebularine's activity as a DNMT inhibitor in other model systems [29], [30], its effect on the expression of DNMTs in HepG2 cells was examined. As expected, zebularine treatment was associated with a statistically significant dose-dependent depletion of DNMT1, DNMT3a, and DNMT3b (Fig. 2A).

Since zebularine decreased DNMT protein levels, to determine whether the growth inhibition and/or apoptosis induction in HepG2 cells by zebularine are a result of a change in DNA methylation, we obtained the genome-wide methylation profiles of zebularine-treated and -untreated (control) HepG2 cells using an Illumina Infinium HumanMethylation450 BeadChip (GEO accession number GSE42490). Among 482,421 assays for CpG sites, 482,260 assays fulfilled our quality control criteria (detection *p* value <0.01 and no missing beta value for both groups) and were subjected to the following analysis. For each assay, delta-beta value ( =  average of the beta values of three zebularine-treated samples – average of those of three controls) was calculated. As shown in Fig. 2B, the methylation profiles were highly similar between zebularine-treated and -untreated HepG2 cells. The number of CpG sites whose delta-beta values are >0.1 and <−0.1 was 35 and 162, respectively. At the majority (99.96%) of CpG sites, methylation levels were nearly the same under the two conditions. To further assess whether these minor methylation changes are observed at specific genes or genomic regions, we conducted region-level methylation analysis using the IMA package [28]. Among 26,659 CpG islands (CGIs), only five showed a significant change (adjusted *p* value <0.05 and |delta-beta value| >0.1) of the methylation level upon zebularine treatment (Table S1). All five CGIs were found to be highly methylated in control HepG2 cells (beta value >0.8), and to be partially hypomethylated (delta-beta range −0.11–−0.21) in zebularine-treatment cells. One CGI is located in an intron of the AGAP1 gene that encodes ArfGAP with GTPase domain, ankyrin repeat, and PH domain 1 protein. Another CGI is located 10 kb downstream of the USP18 gene that encodes ubiquitin specific peptidase 18. The other three CGIs are not associated with any RefSeq gene structure (within 50 kb distance). It is unlikely that the slight decrease in DNA methylation at these five CGIs causes growth arrest and apoptosis in HepG2 cells. These results suggest that the administration of zebularine has little effect on DNA methylation in HepG2 cells, and that the inhibited cell growth and induced apoptosis observed in HepG2 cells upon zebularine treatment are caused by unknown mechanisms that are independent of DNA methylation.

### Zebularine inhibited CDK and phosphorylation of protein retinoblastoma

**Figure 3:**
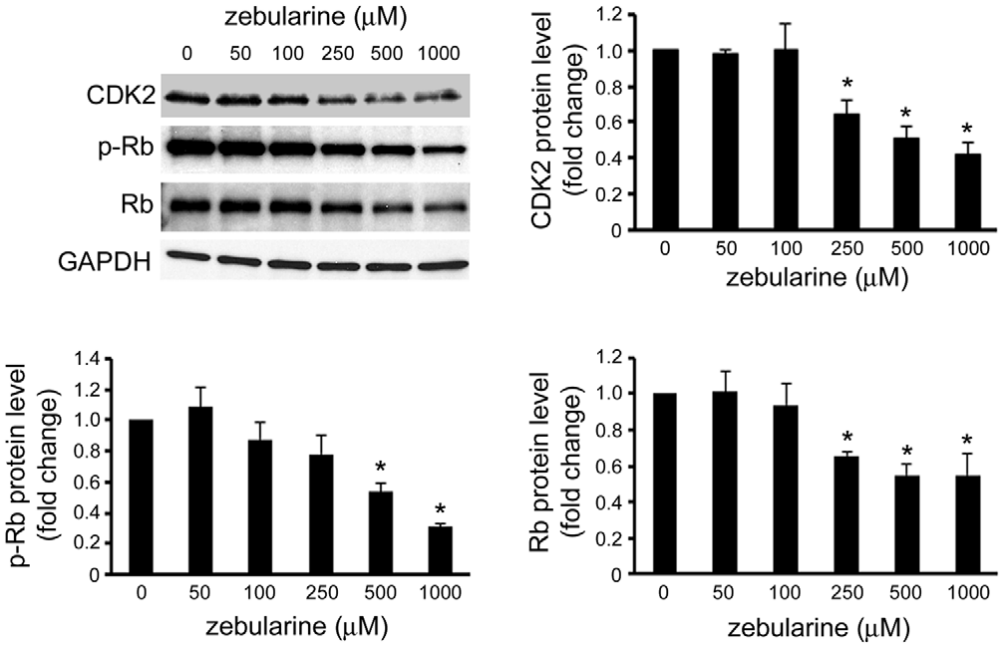
Effects of zebularine on the protein expression of cell-cycle regulator. The protein level of CDK2, p-Rb, and Rb after zebularine treatment for 24 h at different concentrations. After treatment, the cells were harvested and western blot analysis was performed to detect the protein level of CDK2, p-Rb, and Rb. GAPDH was used as a loading control. Data are the means ± SEM of results from at least three independent experiments. *p<0.05, compared to 0 μM.

To estimate the mechanism by which zebularine inhibits HepG2 cell proliferation, we investigated the change in CDK2 expression that was associated with cell-cycle regulation after zebularine treatment. Our results showed that the levels of CDK2 were downregulated in HepG2 cells at 24 h by zebularine treatment (Fig. 3). Protein retinoblastoma (Rb) plays a critical role in governing cell-cycle progression, especially for the transition from the G1 to the S phase [31], where the total and phosphorylation level of Rb was detected. Our results revealed that phosphorylated Rb (p-Rb) decreased in a concentration-dependent manner 24 h after zebularine treatment, which was accompanied by a reduction in total Rb (Fig. 3).

### Zebularine increased p21^WAF/CIP1^ and p53 level in HepG2 cells

**Figure 4:**
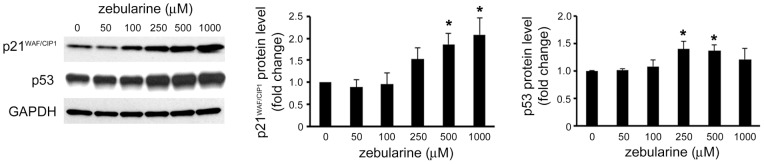
Effects of zebularine on the protein expression of p21^WAF/CIP1^ and p53. The expression of p21^WAF/CIP1^ and p53 after zebularine treatment for 24 h at different concentrations. After treatment, the cells were harvested and western blot analysis was performed to detect the protein level of p21^WAF/CIP1^ and p53. GAPDH was used as a loading control. Data are the means ± SEM of results from at least three independent experiments. *p<0.05, compared to 0 μM.

Previous studies have demonstrated that tumor suppressor protein p21^WAF/CIP1^ and p53 play an important role in G0/G1 arrest in HepG2 cells [32]. Therefore, in order to determine whether these two proteins play a role in inhibiting cell proliferation, the HepG2 cells were exposed to zebularine and analyzed for change on the protein level of p21^WAF/CIP1^ and p53. The results showed that after 24 h of zebularine treatment, the p21^WAF/CIP1^ and p53 protein level was higher in HepG2 cells than in the control (Fig. 4).

### The effect of zebularine on p44/42 MAPK expression

To further clarify the mechanism of the proliferation inhibitory effect of zebularine on HepG2 cells, we examined the expression of p44/42 MAPK in HepG2 cells after zebularine treatment. As shown in Fig, 5, zebularine increased the level of phosphorylated p44/42 MAPK, whereas total p44/42 MAPK was unaffected by the zebularine treatment, as judged by comparisons with GAPDH as a loading control. This data indicates that zebularine can increase the phosphorylation of p44/42 MAPK.

**Figure 5:**
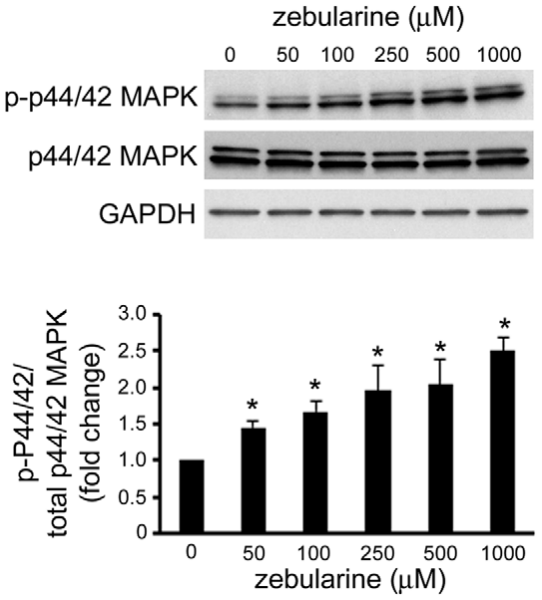
Effects of zebularine on phosphorylation of p44/42 MAPK. The phosphorylation and expression of p44/42 MAPK after zebularine treatment for 24 h at different concentrations. After treatment, the cells were harvested and western blot analysis was performed to detect the phosphorylated and total p44/42 MAPK protein level. GAPDH was used as a loading control. Data are the means ± SEM of results from at least three independent experiments. *p<0.05, compared to 0 μM.

### Zebularine induced apoptosis via caspase pathway

**Figure 6:**
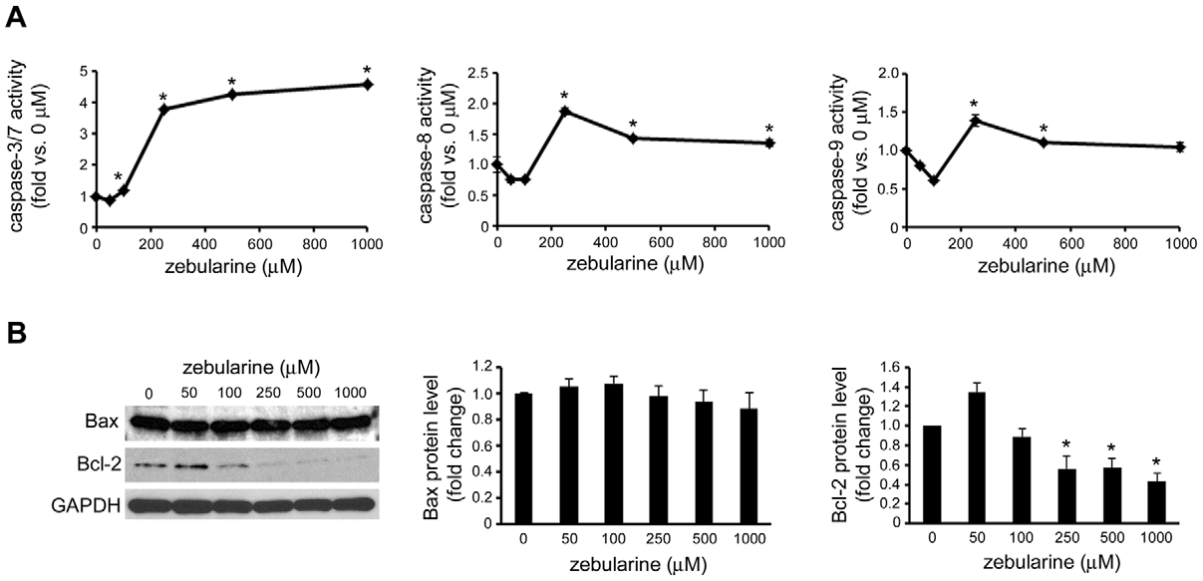
The effect of zebularine on apoptosis-related proteins. HepG2 cells were treated with zebularine at indicated concentrations for 72 h. (A) Caspase-3/7, -8, and -9 activities were determined using Caspase-Glo Assays. The data are expressed as fold-increase relative to the respective untreated samples (RLU/60 min/μg protein). (B) The protein level of Bax and Bcl-2 after zebularine treatment for 72 h at different concentrations. After treatment, the cells were harvested and western blot analysis was performed to detect the protein level of Bax and Bcl-2. GAPDH was used as a loading control. Data are the means ± SEM of results from at least three independent experiments. *p<0.05, compared to 0 μM.

To investigate whether zebularine-induced apoptosis was associated with the caspase family proteins, the activity of caspase-3/7, -8, and -9 was examined after zebularine treatment at 72 h. As shown in Fig. 6A, the activity of caspase-3/7 was significantly increased at an apoptosis-inducible concentration of zebularine. In addition to caspase-3, the activity of caspase-8 and -9 was also increased with zebularine treatment. The expression of the proapoptotic factor Bax and the antiapoptotic factor Bcl-2 was examined by western blotting. The result demonstrated that Bax expression was not affected. On the other hand, Bcl-2 expression decreased with an increasing amount of zebularine (Fig. 6B).

### Zebularine decreases the activity of PKR in HepG2 cells

**Figure 7:**
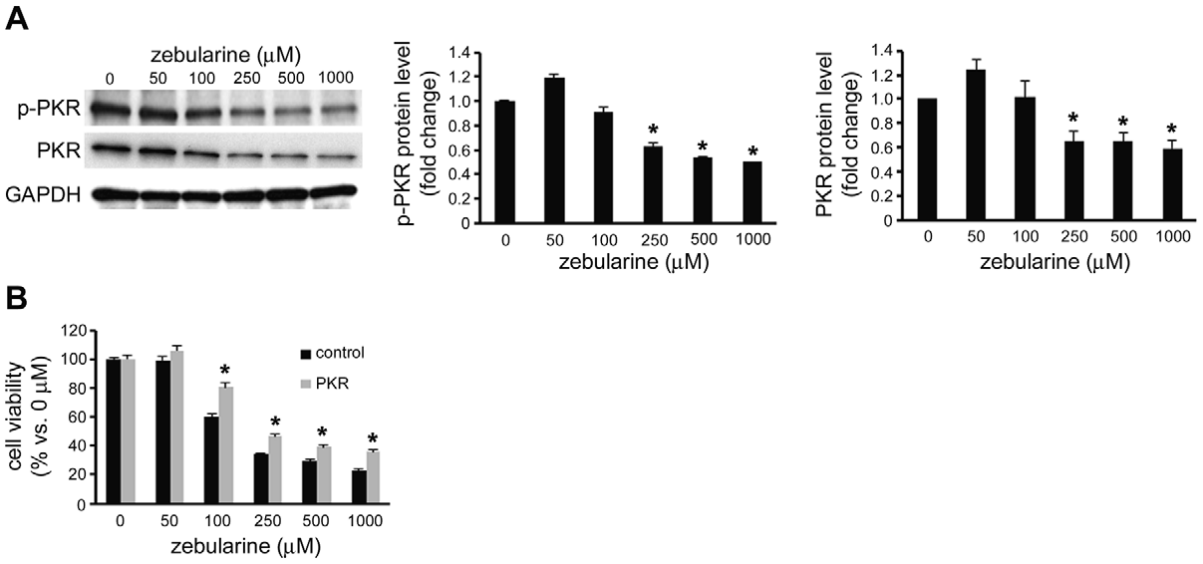
Effects of zebularine on phosphorylation of PKR. (A) The phosphorylation and expression of PKR after zebularine treatment for 72 h at different concentrations. After treatment, the cells were harvested and western blot analysis was performed to detect the phosphorylated and total PKR protein level. GAPDH was used as a loading control. *p<0.05, compared to 0 μM. (B) Effect of the overexpression of PKR in zebularine-induced cell death. The forward transfection of the empty vector (Halo Tag control vector) as the control or the plasmid-containing PKR cDNA sequence (pFN21A-hPKR) was performed, and the cells were then treated with different concentrations of zebularine for 72 h. *p<0.05, compared to control. Data are the means ± SEM of results from at least three independent experiments.

A previous study showed that PKR regulates the protein expression level and phosphorylation of Bcl-2 and plays an anti-apoptotic role in HepG2 cells [33]. Since zebularine can reduce the Bcl-2 protein level, we examined PKR and the phosphorylated PKR level with zebularine treatment. Our results showed that zebularine can reduce the phosphorylated PKR level; this was accompanied by a reduction in total PKR (Fig. 7A). To determine whether PKR has an anti-apoptotic effect in HepG2 cells treated with zebularine, we overexpressed the PKR gene in HepG2 cells and exposed the cells to zebularine. We found that zebularine-induced cell death was reduced by overexpression of PKR (Fig. 7B).

### The effect of zebularine on the activity of PKR in other cancer cells

**Figure 8:**
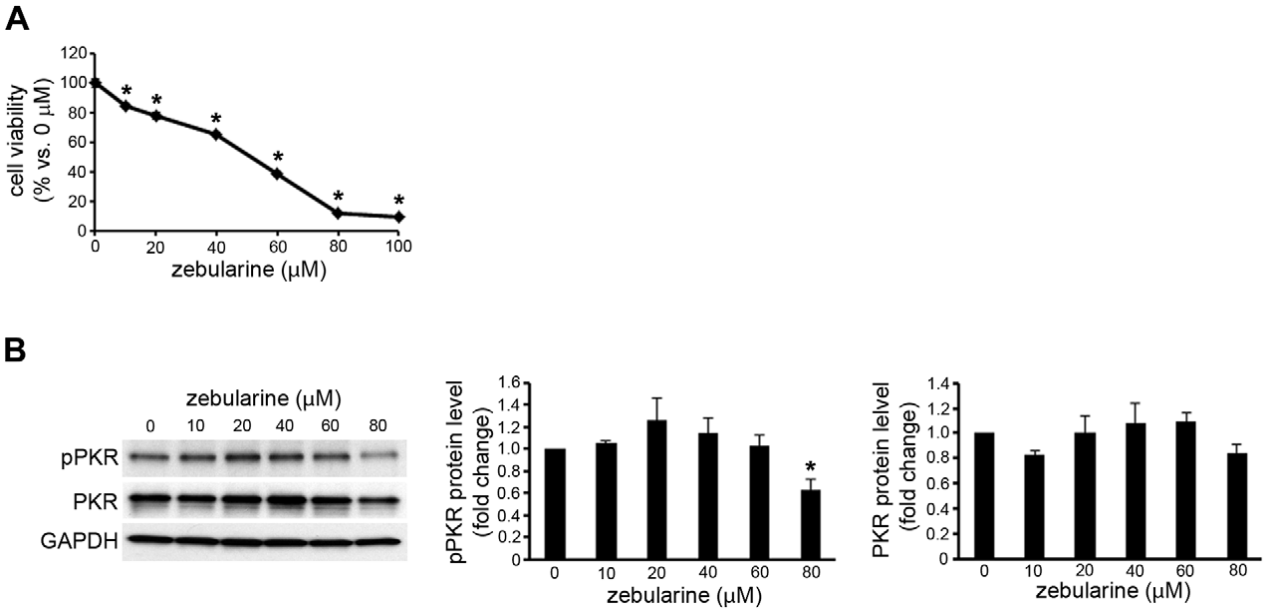
Effects of zebularine on phosphorylation of PKR in HeLa cells. (A) HeLa cells were treated with zebularine at indicated concentrations for 72 h. Cell growth was measured by WST assay. (B) The phosphorylation and expression of PKR after zebularine treatment for 72 h at different concentrations. After treatment, the cells were harvested and western blot analysis was performed to detect the phosphorylated and total PKR protein level. GAPDH was used as a loading control. *p<0.05, compared to 0 μM.

Zebularine also inhibits the growth of bladder cancer, breast cancer, and cervical cancer cells [29], [30], [34]. Since PKR is ubiquitously expressed, we examined whether zebularine decreases the activity of PKR in other cancer cells. It was recently reported that zebularine inhibits the growth of HeLa cervical cancer cells via cell-cycle arrest and caspase-dependent apoptosis [30]. We also observed that zebularine inhibited the growth of HeLa cells, which coincided with the results of the previous study (Fig. 8A). However, our results showed that cell growth inhibiting concentration of zebularine did not reduce the phosphorylated PKR and total PKR levels in HeLa cells (Fig. 8B).

## Discussion

In the present study, we investigated the effect of zebularine on human hepatic carcinoma cells and the possible mechanism. To the best of our knowledge, this is the first study to demonstrate that zebularine inhibits hepatic carcinoma cell HepG2 proliferation by inducing cell growth arrest and apoptosis via intrinsic and extrinsic apoptotic pathways.

In this study, we observed that zebularine decreased the level of DNMT1, DNMT3a, and DNMT3b in HepG2 cells. These results were similar to the reports that DNMT inhibitor induces the depletion of DNMT1, 3a, or 3b protein in human bladder, breast, and cervical cancer cells [24], [30], [35]. Because tight covalent complexes of zebularine and DNMT could lead to compositional change in DNMT protein, it is plausible that DNMTs can be degraded via a ubiquitination system, consequently being observed in the reduction of its expression [30]. On the other hand, our results suggest that zebularine has little effect on DNA methylation in HepG2 cells. Thus, it seems that the cell-cycle arrest and apoptosis observed in HepG2 cells upon zebularine treatment are caused by mechanisms that are independent of DNA methylation.

Eukaryotic cell proliferation is a highly regulated system that is controlled by CDK-cyclin complexes. The cell-cycle transition from the G1 to the S phase was the major regulatory checkpoint in this process. This transition is characterized by the phosphorylation of Rb, and the CDK-cyclin complex catalyzes the reaction [36], [37]. In this study, we found that zebularine inhibited the CDK2 and p-Rb accompanied by a decrease in total Rb, which resulted in cell-cycle arrest and the exertion of its antiproliferative effect. Cell-cycle inhibitor p21^WAF/CIP1^ plays an important role in the G1/S progression process. It may inhibit the activity of the CDK-cyclin complex to regulate cell-cycle progression. These effects can be mediated through p53-dependent or -independent machinery according to the types of stimuli [38]–[43]. There are two p53-binding elements located at the p21^WAF/CIP1^ gene promoter that can be transactivated by the accumulated nuclear p53 after DNA damage [44]. It is reported that p53-dependent G1 growth arrest is mediated by p21^WAF/CIP1^, and p21^WAF/CIP1^ is the CDK inhibitory protein transcriptionally regulated by p53 [45]. Our results showed that the p21^WAF/CIP1^ level was increased after zebularine treatment. In addition, zebularine also upregulated p53 protein. Thus, in the present study, both p53 and p21^WAF/CIP1^ may perform their function by inhibiting the kinase activities of CDK-cyclin complexes to stimulate cell-cycle arrest, which was attributed to the zebularine effect.

MAPKs are essential components of the intracellular signal transduction pathways that regulate cell proliferation and apoptosis. One subgroup of MAPKs, p44/42 MAPK (ERK1/2), is an important target in the diagnosis and treatment of cancer and has been reported to be required for the upregulation of p21^WAF/CIP1^ that results in cell-cycle arrest [46]–[48]. Furthermore, the high-intensity p44/42 MAPK signal leads to the repression of CDK2 kinase activity for p-Rb, which mainly regulates the proliferation of HepG2 cells [49]. In the present study, MAPK signaling pathway regulation after zebularine treatments was investigated. We found that zebularine treatment upregulated the phosphorylation of p44/42 MAPK. Therefore, it is suggested that the p44/42 MAPK pathway plays a role in zebularine-induced cell-cycle arrest by regulating the activity of p21^WAF/CIP1^ and Rb.

During the process of apoptosis, caspases are essential for the initiation and execution of cell death in a self-amplifying cascade in response to various stimuli [50]. Two major apoptotic pathways have been identified: the extrinsic and intrinsic apoptotic pathways. The extrinsic pathway is activated by death receptors, which recruit initiator caspase-2, -8, or -10 through adaptor molecules, whereas the intrinsic signals result in the activation of caspase-9. These initiator caspases can sequentially cleave and activate the effector caspase (caspase-3, -6, and -7), which play an important role in mediating cellular destruction [51]. Our results showed that zebularine appeared to induce the apoptosis of HepG2 cells via the intrinsic pathway, as shown by the activation of caspase-9, and the extrinsic pathway, as shown by the activation of caspase-8, which led to caspase-3 activation. Proteins from the Bcl-2 family can be divided into two groups: suppressors of apoptosis (e.g., Bcl-2, Bcl-XL, and Mcl-1) and activators of apoptosis (e.g., Bax, Bok, Hrk, and Bad). These proteins are key regulators of the intrinsic pathway of apoptosis, setting the threshold for engagement into the death machinery [52], [53]. Among these, the anti-apoptotic Bcl-2 protein acts to suppress apoptosis by preventing the release of apoptogenic proteins, such as cytochrome c, that reside in the intermembrane space of mitochondria. Functionally, Bax acts in opposition to Bcl-2 and facilitates the release of these mitochondrial apoptogenic factors by translocation and oligomerization [54]–[56]. Thus, the ratio of Bax/Bcl-2 determines, in part, the susceptibility of cells to death signals and might be a critical factor in a cell's threshold for apoptosis [57]. In this study, the expression of Bax and Bcl-2 proteins in zebularine-treated HepG2 cells was examined by western blot assay. We found that although Bax protein levels were not affected, Bcl-2 protein level was downregulated with zebularine treatment, which led to a marked increase in the Bax/Bcl-2 ratio and then apoptosis.

Initially identified as an antiviral protein, PKR is best known for triggering cell defense responses and initiating innate immune responses by arresting general protein synthesis and inducing apoptosis during virus infection [58]. Activated PKR, known as a eukaryotic initiation factor 2-alpha (eIF-2α) kinase, induces the phosphorylation of eIF-2α [59], which inhibits the initiation of translation through the tRNA-40S ribosomal subunit. On the other hand, PKR is involved in controlling the transcription of Bcl-2 in HepG2 cells, mediated by the transcription factor NF-κB [33]. In this study, we observed that zebularine can reduce the phosphorylation of PKR, which indicates the activated PKR. In addition, overexpression of PKR reduced zebularine-induced cell death. Thus, our results suggest that zebularine decreases the activity of PKR and results in apoptotic cell death via reduced NF-κB activity and the downregulation of Bcl-2. The fact that zebularine inhibits the growth of bladder, breast, and cervical cancer cells [29], [30], [34] and that PKR is ubiquitously expressed led us to hypothesize that zebularine induced the cell growth arrest via the downregulation of PKR in other cancer cells. When we examined the effect of zebularine on PKR expression in HeLa cells, we observed, however, that zebularine did not decrease the phosphorylation of PKR and the total PKR level. These results suggest that there are differences in the mechanism by which zebularine inhibits cell growth among the different types of carcinomas. The action and mechanisms of zebularine must therefore be further investigated in other cancer cells.

In conclusion, our observation indicated that zebularine inhibited cell growth and induced apoptotic cell death, which contributed to its antiproliferation effects against hepatocellular carcinoma HepG2 cells. The most likely mechanism underlying the zebularine-induced growth arrest involves an initial induction of p44/42 phosphorylation and an increase in p21^WAF/CIP1^ expression, which leads to a reduction in G1-related CDKs such as CDK2 protein and p-Rb, and then ultimately arrests the HepG2 cell cycle. Furthermore, zebularine decreased the activity of PKR, and resulted in apoptotic cell death via the downregulation of Bcl-2.

## Supporting Information

Table S1 **List of CGIs showing a significant change in DNA methylation level upon zebularine-treatment in HepG2 cells.**
(XLS)
